# Role of multimodal imaging in the preparation for surgery to correct left atrial myxoma

**DOI:** 10.1002/hsr2.215

**Published:** 2020-12-03

**Authors:** Flor Baeza Garzón, María Manuela Izquierdo Gómez, Mauro Andrés Di Silvestre Alonso, Juan Lacalzada‐Almeida

**Affiliations:** ^1^ Hospital Universitario de Canarias La Laguna Tenerife Spain

The patient was a 60‐year‐old woman with rheumatoid arthritis referred for dyspnea. Transthoracic echocardiography (TTE) revealed a mass occupying the left atrium. 3D transesophageal echocardiography (TEE; Figure 1A, arrow) reveal the mass had prolapsed into the left ventricular inflow tract without adhering to the mitral valve, although it led to hemodynamically significant stenosis but the mass was adhered to the atrial septum via a broad stalk. These findings pointed to a diagnosis of myxoma. Computed tomography revealed a large mass attached to the interatrial septum with a broad stalk and an afferent artery dependent on the circumflex coronary artery (Figure 1B, arrowhead). A mass measuring 75 × 60 × 60 mm (Figure 1C) was removed during surgery, with wide resection of its base of implantation. Given the large size of the pedicle (Figure 1C, arrow), this necessitated removal of a large part of the interatrial septum and subsequent closure of the septal defect. Histopathology confirmed the diagnosis of myxoma. Outcome was favorable. A follow‐up TEE revealed mild mitral insufficiency. Various studies have shown the value of multimodal imaging in the diagnosis and surgical management of myxoma. While cardiac magnetic resonance is the gold standard, TTE is the first‐line diagnostic technique,^1^ with 2D and 3D TEE being the approaches that best define the morphology and location of the stalk. This is important in the differential diagnosis and in planning surgery,^2^ since these techniques make it possible to evaluate the correct functioning of the mitral valve and identify the exact location and size of the base of implantation. These data are essential for deciding on the best access to the cavities and for predicting resection and reconstruction of the structures. Computed tomography was useful for locating and evaluating the tumor vasculature and ruling out coronary disease.^3^, ^4^

**FIGURE 1:**
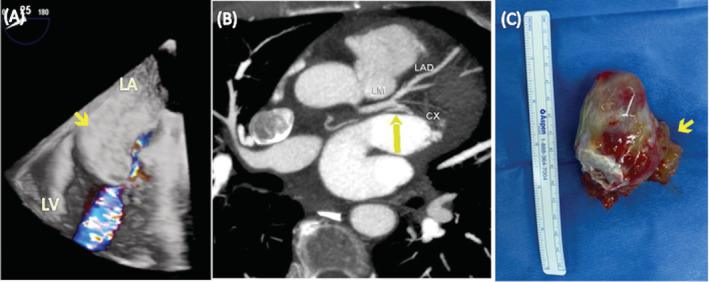
A, Narrow: 3D transesophageal echocardiography reveal the mass had prolapsed into the left ventricular inflow tract without adhering to the mitral valve. B, Computed tomography revealed a large mass attached to the interatrial septum with a broad stalk and an afferent artery (arrowhead) dependent on the circumflex coronary artery. C, Mass measuring 75 × 60 × 60 mm was removed during surgery with his pedicle (arrow)

## CONFLICT OF INTEREST

The authors declare no conflicts of interest.

## AUTHOR CONTRIBUTIONS

Conceptualization: Flor Baeza‐Garzón, Juan Lacalzada‐Almeida

Resources: Flor Baeza‐Garzón, Mauro Andrés DiSilvestre Alonso

Supervision: María Manuela Izquierdo Gómez

Validation: Flor Baeza‐Garzón, Juan Lacalzada‐Almeida

Visualization: Flor Baeza‐Garzón, Juan Lacalzada‐Almeida

Writing—Original Draft Preparation: Flor Baeza Garzón, Juan Lacalzada‐Almeida, Mauro Andrés DiSilvestre Alonso

Writing—Review & Editing: María Manuela Izquierdo Gómez

  All authors have read and approved the final version of the manuscript.

  Corresponding author had full access to all of the data in this study and takes complete responsibility for the integrity of the data and the accuracy of the data analysis.

## TRANSPARENCY STATEMENT

The lead author (Flor Baeza Garzón, MD, PhD) affirms that this manuscript is an honest, accurate, and transparent account of the clinical image being reported; that no important aspects of the case have been omitted.
